# Proposed Implementation of Blockchain in British Columbia’s Health Care Data Management

**DOI:** 10.2196/20897

**Published:** 2020-10-23

**Authors:** Danielle Cadoret, Tamara Kailas, Pedro Velmovitsky, Plinio Morita, Okechukwu Igboeli

**Affiliations:** 1 Science and Business Program Faculty of Science University of Waterloo Waterloo, ON Canada; 2 School of Public Health and Health Systems University of Waterloo Waterloo, ON Canada; 3 Institute of Health Policy, Management, and Evaluation University of Toronto Toronto, ON Canada; 4 Research Institute for Aging University of Waterloo Waterloo, ON Canada; 5 Department of Systems Design Engineering University of Waterloo Waterloo, ON Canada; 6 eHealth Innovation Techna Institute University Health Network Toronto, ON Canada

**Keywords:** blockchain, electronic medical records, health data management, patient centric

## Abstract

**Background:**

There are several challenges such as information silos and lack of interoperability with the current electronic medical record (EMR) infrastructure in the Canadian health care system. These challenges can be alleviated by implementing a blockchain-based health care data management solution.

**Objective:**

This study aims to provide a detailed overview of the current health data management infrastructure in British Columbia for identifying some of the gaps and inefficiencies in the Canadian health care data management system. We explored whether blockchain is a viable option for bridging the existing gaps in EMR solutions in British Columbia’s health care system.

**Methods:**

We constructed the British Columbia health care data infrastructure and health information flow based on publicly available information and in partnership with an industry expert familiar with the health systems information technology network of British Columbia’s Provincial Health Services Authorities. Information flow gaps, inconsistencies, and inefficiencies were the target of our analyses.

**Results:**

We found that hospitals and clinics have several choices for managing electronic records of health care information, such as different EMR software or cloud-based data management, and that the system development, implementation, and operations for EMRs are carried out by the private sector. As of 2013, EMR adoption in British Columbia was at 80% across all hospitals and the process of entering medical information into EMR systems in British Columbia could have a lag of up to 1 month. During this lag period, disease progression updates are continually written on physical paper charts and not immediately updated in the system, creating a continuous lag period and increasing the probability of errors and disjointed notes. The current major stumbling block for health care data management is interoperability resulting from the use of a wide range of unique information systems by different health care facilities.

**Conclusions:**

Our analysis of British Columbia’s health care data management revealed several challenges, including information silos, the potential for medical errors, the general unwillingness of parties within the health care system to trust and share data, and the potential for security breaches and operational issues in the current EMR infrastructure. A blockchain-based solution has the highest potential in solving most of the challenges in managing health care data in British Columbia and other Canadian provinces.

## Introduction

### Background

Blockchain is a relatively new technology with the potential to revolutionize the way data are secured and managed. Implementing blockchain in health care opens diverse possibilities in data analytics, cross-province health data transfer, and patient-centered health care. It also helps resolve many of the existing challenges faced in health care data management, such as eliminating information silos, increasing efficiency in data transfer between health care facilities, data sharing, and data protection [1,2]. Challenges associated with blockchain implementation in the Canadian health care system include complicated regulations surrounding health and personal data, the government’s universal involvement, and transitional issues between 2 information systems. Identifying gaps in the current infrastructure and the problems that blockchain can solve and creating a realistic implementation plan for blockchain systems in the Canadian health care system will require an in-depth strategic analysis that will inform viability [1,3].

### What Is Blockchain?

Blockchain technology is a distributed ledger system that can be used for the management and exchange of information among members of a network [4,5]. Each participating computer in the network forms an independent node that maintains a copy of the ledger, which is automatically updated among all members whenever there is an addition to the ledger.

A transaction in the network is timestamped and cryptographically sealed in a *block* and then added to the chain of previous blocks (representing previous transactions) by an automated validation mechanism [4]. When a block is added to the chain, a cryptographic technique called a hash is used to ensure that the connection is immutable. Hashes are one-way digital *signatures* comprising a string of numbers and letters, and each hash is based on the hash of the preceding block. Hashes are what give blockchain its immutability, an important feature that prevents malicious changes to the blockchain [4].

Another cryptographic technique, zero knowledge proof (ZKP) systems, is often combined with blockchain to reduce the amount of data needed to be shared between parties. ZKPs are interactive proofs that have the ability to yield nothing but the validity of a claim [6-8]. A public blockchain can be joined by anyone, whereas a permissioned and private blockchain requires permission from the governing nodes of the network to join, thus upholding trust and security of the network [4]. The term *distributed* refers to the way that the blockchain or ledger is stored in the network: rather than being stored in 1 database, a copy of the blockchain is stored with each member of the network [4]. It is important to indicate that this study focuses on the use of private blockchains; public blockchains are not built to accommodate or safeguard sensitive information properly (eg, transactions involving medical records) and, as such, are not a viable solution for this scenario.

### Current Challenges in Health Care

Electronic medical records (EMRs) have a large potential to benefit from blockchain systems. Information in EMRs may include patient diagnoses and histories as well as test and imaging results. However, the fragmentation of digital data occurs when various points of care use different EMR systems to store patient data, which are not interoperable [9]. Patients visit numerous health care offices throughout their lives, leading to fragmented medical records, which in turn limit the information available to practitioners and service providers. Ultimately, this affects the quality of care received by the patients and patients’ experience with the health care system. It becomes more difficult for health care practitioners to see the complete picture of a patient’s care, and this often results in redundant procedures and history taking, which takes a toll on the health care system and the patient.

Data sharing between care providers improves the accuracy of diagnoses [10-12] and reduces errors in treatment plans [13]. In addition, with telemedicine on the rise, there’s a need to identify a way that allows efficient and secure sharing of patient data and consent [14]. Currently, there are no existing interoperable EMRs as a pan-Canadian endeavor involving the support and collaboration of federal and provincial governments to minimize the presence of information silos. Each province and territory is responsible for developing its own EMR strategy, leading to a wide range of differences in EMRs throughout Canada, which reduces interoperability [1,2,15].

In terms of national efforts to increase interoperability between EMRs, the federal government issued a Can $1.6 billion (US $1.2 billion) grant in 2001 to establish the Canada Health Infoway (Infoway), an agency specifically formed to spearhead the EMR initiative [16]. Infoway partnered with the Canadian Institute for Health Information (CIHI), an existing agency working to improve Canadian health care based on informatics, to establish national standards and guidelines for provinces to follow in their EMR adoption [17]. Despite both these agencies working together, the problem of interoperability prevails both within and across provinces. This is partly because of the variability in government-approved EMRs, which prevents complete standardization [16].

The medium in which patient health data are stored varies across provinces, regions, and institutions. These data can be stored as paper records or EMRs, which can generally be referred to as a point-of-care service applications (PCSAs). At this point, data are stored at an institutional level wherein accessibility to external parties is limited [18]. External parties may include other medical clinics, hospitals, or institutions. CIHI outlines that data stored in the PCSA may feed into the integrated assessment record (IAR). The IAR is a tool that enables a centralized repository for patient health data collected from separate centers of care with differing PCSAs [18]. Ideally, physicians and health professionals from various institutions can access the IAR, allowing interoperability. However, successful implementation and adoption of the IAR are not well documented for health departments outside long-term care, mental health care, or community health and support services [18]. These sectors capture only a portion of the required health services and do not span the Canadian health care population. Two variables that contribute to the limited success of IARs are the lack of digitization of paper health records and the lack of standardization for PCSAs.

In addition, although new technology attempts to solve these issues, there is also the problem of physicians’ resistance to change [19]. Many health care professionals do not trust computers and tablets to store information because they feel it is not as reliable as a pen and paper. Data loss, computer malfunctions, and breaches of privacy are often cited by those protesting against digitizing information. Furthermore, when hospital administrations decide to implement an EMR, many health care professionals do not adopt the software correctly and continue using their paper charts and dictaphones. This leads to a strain on time and resources and increases redundancy and errors.

Finally, a major issue is the ownership of medical records. Currently, patient records are either owned by the hospital or by the doctor in a private practice setting, and the onus is on these parties to keep records private and safe. With a large amount of evidence showing that patient-centric health care improves outcomes, there is a push toward letting patients access and own their records. This gives patients the ability to give full or partial controlled access to their records whenever and to whomever they want. However, patient-centric health care is impossible with the current health care data infrastructure. EMRs were simply not designed with the patient in mind, and their workflows and interfaces completely exclude the patient. Flipping the current infrastructure around to accommodate patient-centric health care would require completely redesigned systems.

### Goal of the Study

This study aims to provide a detailed overview of the current *status quo* of the health data management infrastructure in British Columbia to identify gaps and inefficiencies in the system. This review explores whether blockchain is a viable option for the existing gaps in EMR solutions in the Canadian health care system. This study aims to analyze the benefits of a blockchain-based data solution as well as the feasibility of switching to this technology. The expected results of this study include an implementation plan for a blockchain-based solution, which can guide future parties interested in moving forward toward a more universal, efficient, and integrated patient-centric health care data management system.

## Methods

### Flow of Health Data in British Columbia

The British Columbia health care data infrastructure was chosen as a model for blockchain implementation in this study because of the availability of an industry expert who provided insight into the nuances of British Columbia’s structure and processes. This expert is well informed of the health systems information technology (IT) infrastructure of British Columbia’s Provincial Health Services Authorities (PHSAs) and is knowledgeable of the realities of health data management. This consultation supplemented details regarding the *status quo* and health information flow within the province that would not have been accessible to public domains. Information flow gaps, inconsistencies, and inefficiencies were the target of the analyses.

An important metric to measure the viability of a blockchain solution is the digitization rate of paper records. Blockchain requires digital data; without digitized records, a blockchain system cannot be implemented.

### Blockchain and Existing Solutions

Existing technological solutions for health care data management include the following:

1. IT solutions: rely on host EMR systems that allow health data to be stored on local computers (designated servers). The hosted EMR system allows a facility to take ownership of their health data [20].
2. Cloud computing: uses a network of remote servers that store and manage information. Unlike onsite storage of information or physical paper charts, data are stored outside the health facility with cloud computing [20].
3. MedRec: an existing blockchain-based EMR system developed by the Massachusetts Institute of Technology (MIT) that stores references to the off-chain location of medical records [2,21,22].

The integration of blockchain with these existing solutions and Canada’s existing health care data infrastructure was explored using published literature and consultations with health IT and blockchain domain experts.

The implementation plan was designed on the basis of this information. It focused on bridging the gaps in British Columbia’s current infrastructure and easing pain points of patients, health care professionals, and IT personnel. It aims to move toward a blockchain-based, universal, patient-centric health care data management system in Canada, taking existing infrastructure and ongoing health data needs into account.

## Results

### Digitization and the EMR Status Quo in British Columbia

The Ministry of Health in British Columbia (BCMOH) is the jurisdictional body responsible for EMR implementation. Their goal is to deliver relevant data to health care professionals, putting in place an infrastructure for health information sharing and thus increasing interoperability. The BCMOH established the Physician Information Technology Office (PITO) to launch the transition from paper records to EMRs in British Columbia [23]. The BCMOH’s primary task is to provide systems management and recommend EMR systems for physician offices to effectively replace paper-based records. These are all performed under Infoway’s guidelines to improve interoperability.

Currently, the system development, implementation, and operations of EMRs are carried out by the private sector. In 2007, the BCMOH partnered with Sun Microsystems to build, design, implement, and operate a British Columbia–specific EMR system. Although this has not yet surfaced, the province currently uses 4 government-approved EMR applications:

1. Intrahealth: a web-based IT solution that has various modes of deployment and databases that can be customized for the physicians’ preferences [24].
2. Med Access: a web-based system that configures individual clinics and user preferences. The platform adapts to the workflow of those using it through tailoring based on role, group, and organization. The software is designed to use the internet so that patient information is not stored on the end user’s computer [25]. Beyond acting as an e-chart, the system also allows for point-of-care decision support through reminders and prompts. Furthermore, interprovider communication is available with other caregivers who are also using Med Access EMR [26].
3. Wolf EMR: a cloud-based EMR system that manages and stores patient data in a customizable manner. It allows physicians to create queries and use support tools within each patient file to optimize the care provided [26].
4. Osler Systems: information on this system was not publicly available.

As of 2013, EMR adoption in British Columbia was at 80% across all hospitals [27]. Adoption was defined as the initial implementation of an EMR system in a facility but does not exclude the use of paper records. Consequently, the focus has shifted toward more optimal use of EMRs and achieving interconnectivity across various points of care.

### Flow of Health Data in British Columbia

**Figure 1:**
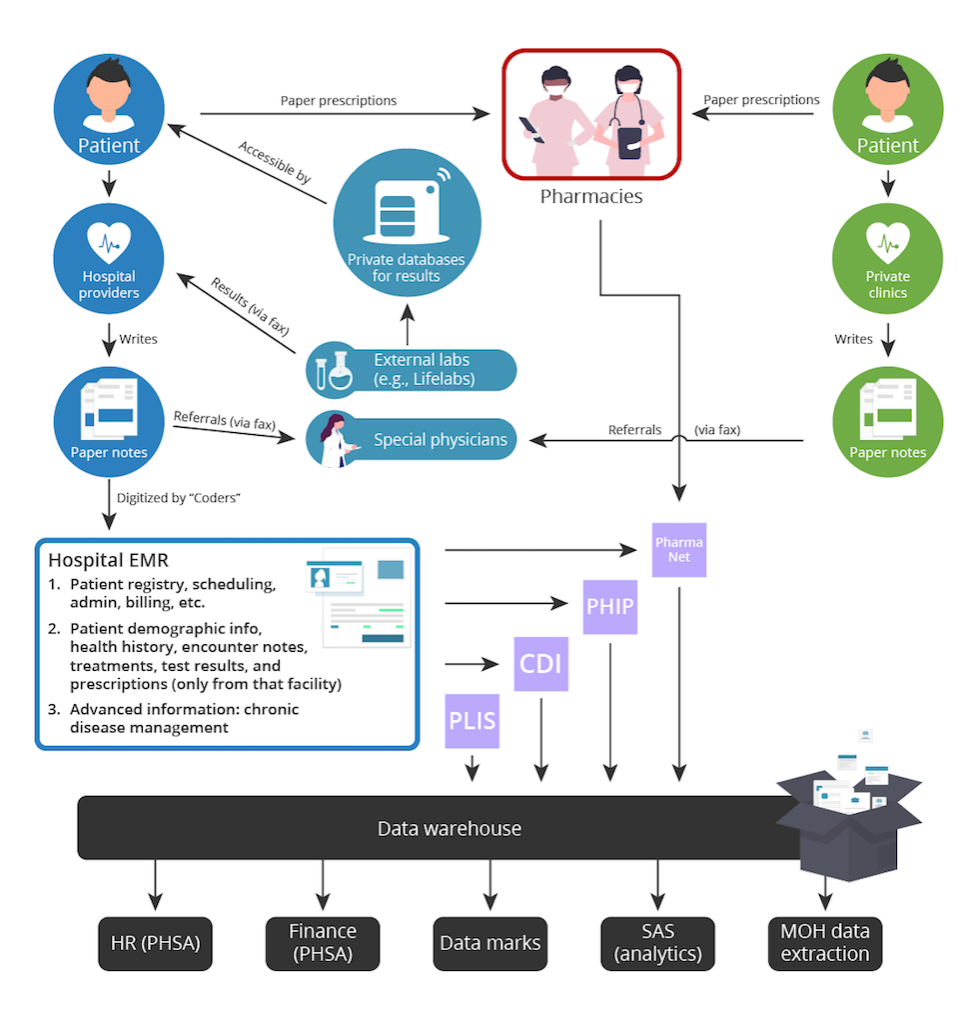
Flow of health care data in British Columbia Provincial Health Services Authority. CDI: Clinical Diagnostic Imaging; EMR: electronic medical record; HR (PHSA): Human Resources (Provincial Health Services Authority); MOH: Ministry of Health; PHIP: Public Health Improvement Plan; PHSA: Provincial Health Services Authorities; PLIS: Provincial Laboratory Information Solution.

A snapshot of the health care information flow in the British Columbia’s PHSA (BCPHSA) is presented in Figure 1. Patient information is introduced to the health care system when an individual visits a hospital or private clinic. These scenarios are indicated by the 2 stars. In a hospital setting, the information flow begins when a patient gives information to health care workers, such as the reason for their visit, demographic information, medical history, symptoms, and more. Patient information gathered from these consultations can then (1) be directly added into an EMR system based on technology integration at a specific hospital or (2) be first recorded on physical paper charts or dictaphones and then transcribed to an electronic format later. In the latter, the process of entering these physical records into the EMR itself could have a lag of up to 1 month. During this lag period, disease progression updates are continually written on physical paper charts and not immediately updated in the system, creating a continuous lag period and increasing the probability of errors and disjointed notes (personal communication, data consultant).

The large blue box on the left depicts a hospital EMR and outlines the multiple functions for which it is responsible (Figure 1). At the basic level, EMRs record hospital admissions, staff, facility scheduling, and billing information. Further functionalities include patient histories, health care staff’s patient notes, treatment information, and test results, among others. Some EMR systems can also provide more advanced information such as drug interaction databases, chronic disease management, and statistics on treatment outcomes. These advanced functions guide physicians to practice evidence-based medicine, an important facet of today’s medical world.

In the BCPHSA, a hospital EMR will send the information it gathers to province-wide databases depicted by the light purple squares (Figure 1), as listed below:

1. PLIS: Provincial Laboratory Information Solution, a database for laboratory test results.
2. CDI: Clinical Diagnostic Imaging, a database for diagnostic imaging results and scans.
3. PHIP: Public Health Improvement Plan, a provincial body that analyzes health information and tries to improve health outcomes with conclusions from these data.
4. PharmaNet: the provincial drug information repository that also connects all pharmacies.

These databases are independent entities that maintain their systems. They are all legally required to send their data to the data warehouse (depicted in black in Figure 1), which is the central storage and integration facility for all health information in the BCPHSA. The data warehouse stores this information, formats it appropriately, and disseminates information to teams that create data marks, which are processed data sets that are ready for analysis. SAS (SAS Institute), an analytics software, is used to produce reports based on the data collected. Finally, the BCMOH requires certain data to be reported periodically, such as hospital admittances, discharge statistics, and so on.

External laboratories are groups that are not associated with the hospital system and are represented by light blue shapes. Some laboratories have their private websites, where both health care personnel and patients can log in to view their test results. Specialist clinics are also represented as external bodies separate from the hospital system or primary care physicians. For these, the flow of information comes in the form of referrals that are often faxed directly from the primary care physician to the specialist, whereas the patients themselves are commonly requested to also bring a physical copy of the referral to make an appointment with the specialist.

Primary care private practices encompass a large portion of the public’s interaction with the health care system. These bodies are represented in green (Figure 1). This is the path through which patients’ health care data go through when they visit their family doctor, walk-in clinics, etc. Information about patients is stored within the private clinic’s own EMR and is not sent to any other facility. According to the industry expert consulted for this work, this is often the case as well for specialist clinics that are not attached to hospitals. Finally, patients and pharmacies are connected only through physical prescriptions. Patients may receive a printed or even handwritten prescription to bring to the pharmacist to collect their medications.

### Alternative Solutions for Health Data Management

Hospitals and clinics have several choices for managing electronic records of health care information, such as EMR software or cloud-based data management. Currently in development are blockchain-based EMR systems such as MedRec, but these systems are only in their testing phases in the United States and in some countries in Asia; they are neither available nor in testing in Canada at the time of publication. Cloud computing, EMR software, and MedRec are discussed in the following paragraphs.

Cloud computing is a well-adopted alternative that uses a network of remote servers to store and manage information. It differs from traditional EMR software in that the data are not tangibly stored within the facility [20]. In theory, cloud-based EMRs can be streamlined and centralized to enable interoperability. However, it is very challenging to do so if there is no additional software integration across all communication facilities, especially if they use different cloud-based EMR systems [20]. The primary benefit of cloud computing is that there is access to reliable network backups and there is a contingency plan for data loss to local hardware failures. Currently, most EMR systems used are cloud based given that these are less rigid and easier to update with new information in comparison with a local host system [20]. According to the Capterra EMR Software Directory, the top 5 cloud-based systems by provider utilization are eClinicalWorks, Allscripts, CureMD, Epic, and Cerner [20]. Therefore, unlike host EMR software, interoperability could be an actual possibility through cloud-based EMR, but it would still be a major challenge [20].

There are hundreds of vendors offering EMR software that relies on an EMR host system. In this solution, all data are stored on one computer, designated as a server that houses a database file [20]. This file contains all the desired data. Every computer in a facility has a workstation installed that is linked to the server and is continuously sending and receiving data from the server. It is important to note with EMR software that although workstations require installation, data from the server can still be accessed outside the facility [20]. One of the main benefits of IT solutions that entail a hosted EMR system is that it allows a facility to take ownership of the data [20]. However, because of the wide selection and range of host EMRs that suit different environments, health care facilities often choose different software that are built very differently and therefore cannot communicate with each other. This makes interoperability and data sharing across points of care nearly impossible.

The blockchain-based EMR platform MedRec, an MIT project, is currently being tested and is thought to be a viable solution for health care data management. It is designed for a patient-centric health care model that focuses on managing authentication, confidentiality, and data sharing [28]. The system is completely decentralized and is based on access rights to a private blockchain. Patients and providers are given control over the access and retrieval of relevant medical records [28]. The system network operates on the internet, but there is no central repository of permissions or private data, which decreases the incentive to hack the system as such an attempt yields a low yield. The system works by establishing a relationship between the patients and the medical record originator on the blockchain. This then creates the foundation of a smart contract that other members of the network must use to request access to the medical record [28]. The medical records themselves are held by the patient in a wallet, accessible on their mobile devices. Smart contracts are executed automatically when the required conditions are met. These smart contracts remove the necessity to trust any one party with the storage and sharing of congregated personal health data.

It is important to note that for nonblockchain solutions, interoperability is the underlying constraint with existing alternatives for health data management. There are administrative and bureaucratic power dynamics at play in selecting EMR software or cloud-based EMR systems that perpetuate the misalignment of systems across entities (personal communication, data consultant). The sunk costs associated with upheaving employed systems and software are reasons for pushing back on the topic of standardization. Although it is not costly to update existing alternative systems and software, it is not cost-effective to replace current systems for a marginally similar alternative (personal communication, data consultant).

### Blockchain for Health Data Management

The current major stumbling block for health care data is interoperability, as health care facilities across Canada use a wide range of unique information systems [29]. However, if blockchain is used to create the overarching health care information system architecture, this issue could be significantly improved. Blockchain can retrieve and make use of any digitized information contained in each system via an application programming interface (API), which is a package of code that teaches the blockchain how to access the data in a different information system [29]. The API method can be used as a transition toward a fully blockchain-based EMR. In this manner, replacing the EMR or data system in each facility can be implemented over a longer period, as each system simply requires an API that connects it to the blockchain. Each facility can continue to use their system that serves their needs, and physicians’ resistance to change, another major issue in health care data management, can be tackled over a much longer grace period of 3 to 4 years. The strain on health systems and the resource requirement needed for the proposed blockchain solution are far less when compared with simply switching EMRs abruptly. Abrupt switches often require a complete change in systems over 6 months to 1 year and often have negative effects on clinic workflows and efficiencies [29]. In addition, immediate costs will be greatly reduced, as installation and training will be more spread out.

Cryptocurrencies are often mentioned in the same breath as blockchain, which may lead to some confusion over why a cryptocurrency does not exist in this study’s application of blockchain. First, it must be understood that blockchain is not the same thing as cryptocurrency [30]. Blockchain is the underlying technology that enables the creation of cryptocurrencies; thus, cryptocurrencies are simply an application of blockchain [30]. In the same vein, the patient-centric, verifiable, and secure health record management system this study suggests is simply another application of blockchain technology. In some applications of blockchain, cryptocurrency can be added as an incentive program [31-33]. For example, in a blockchain-based electric vehicle charging station scheduling program, users are rewarded with coins if they use the charging station during an off-peak period but receive no coins if they use it in a peak period [31]. Therefore, there is a potential to add cryptocurrency to the health care data blockchain if there is a need to incentivize some users to do certain things. For example, you could reward patients who renew their prescriptions on time or those who follow their vaccination schedules properly. However, this incentive program is not vital to the operation of the health care record blockchain; rather, it is more of a bonus than an integral component. As such, a cryptocurrency will not be included in first-generation health care blockchains but may be an option in the future as health care blockchains develop.

One of the biggest features of using a blockchain to store health care data is the possibility of patient-centric health care [34]. Patient-centric health care increases patients’ understanding of their condition and allows the patient to play a more active role in their health care. This has been proven to increase concordance and adherence, improving health outcomes. [35]. Poor adherence to treatment plans costs Canada more than Can $9 billion (US $6.8 billion) per year [35]. In addition, because medical records are attached to the patient and not the provider, it makes seeking care in different provinces or even different countries much easier.

Blockchain also allows a user to have varying degrees of anonymity and privacy from each node in the network [34]. These nodes can represent health care facilities, government agencies, or even individual patients. A critical issue in health care is that different entities do not fully trust each other, which impedes data sharing between entities. This is linked to poorer health outcomes, as mentioned above [10-13]. The nature of blockchain allows for *trustless disintermediation,* which enables parties who do not trust each other to share certain digital information when protecting their private data [36]. In a private blockchain, each node is only allowed to join after their identity is verified, and the majority of the network approves it. For nodes in the network that own medical records, blockchain allows fine control of the specific information that they choose to share with other nodes in the network. If one wishes, relevant records could be shared without revealing any personally identifiable information (PII). This is because PII is never stored on the blockchain itself; it is always on *state channels*, which are interactions that are conducted off the blockchain [34]. In summary, each node will have its personal identity key, an alphanumeric code masking their identity, which is linked to all the medical records they own. This key can be used to retrieve information whenever they want. If patients want to share their medical records, they can conduct a state channel interaction with a health care professional or facility on a state channel [34]. This interaction allows them to transmit the particular records they choose to share with the health care provider, and afterward, a record of this transaction will be published onto the blockchain.

The decentralized manner in which information is stored on a blockchain discourages hacking efforts, as mentioned above when describing MedRec. Currently, personal data are stored in centralized databases. Hacking of this centralized system will leak every single file the database contains, along with all the personal information attached to each record [34]. If Canada decides to build a single, centralized database with the health records of all Canadians on it to enable interoperability, it becomes an immense target for hackers [37,38]. This is a bad idea as demonstrated by examples from around the world: in 2016, 15.5 million EMRs were breached in the United States and the global health care industry spent US $6.2 billion in that year alone to deal with security breaches [37]. The value of a hacked EMR in the black market is high, estimated to be approximately 10 times the value of a credit card number, increasing the incentive to attack EMRs [39].

However, with a decentralized blockchain model, keys can only be hacked individually. With 1 hacked key, the hacker gains access to only a single person’s files and information. There is no large database of information freely available to steal from [34]. If there were health records of 35 million people on the *Canadian Healthcare Data Blockchain*, a hacker would have to hack the blockchain 35 million times to gain access to the entire database. In addition, to be able to manipulate the network, the hacker must attack 51% of all computers, which is currently infeasible without prohibitively large computer resources [3].

### Centralization Versus Decentralization in a Blockchain Context

To further explore whether centralization or decentralization is more beneficial for Canada’s health data management, definitions are required. Current solutions for interoperability lead to a centralized solution. This means creating a centralized repository of all the data from different health care bodies and allowing certain entities access as required. This allows for a certain degree of interoperability, as multiple entities can access data in that central repository. A few of these central repositories already exist, as presented in Figure 1, such as PLIS, CDI, PHIP, and PharmaNet. However, this has not solved interoperability issues at the point-of-care level, which negatively affects patient care and therefore affects health outcomes, as discussed above [10-13]. Critical issues such as data security of a giant central repository [3,34], determining access rights for different clinics or institutions, and a general lack of trust between most health care organizations and clinics severely limit the centralization solution (these issues are further discussed in the Results section). In addition, ownership of the data also becomes an issue. Who will be the owner of all this information? How will the owner or controllers of the repository be chosen? What institution will be held responsible if there is a data leak or if data are lost? This lack of trust among different health care bodies hinders willingness to hand over patient data and willingness to allow others to access their patient data. It also makes it unlikely that organizations will be able to cooperate sufficiently to maintain a centralized repository together.

However, blockchain solves the issue of interoperability without introducing centralization. Blockchain allows each body to store their data in their respective *node,* and data are then accessed upon request from a connecting node. There is neither a centralized node that stores all the information nor a central body that owns or controls the whole network [4]. Therefore, the blockchain acts as a network that allows independent bodies to communicate when retaining their desired level of privacy and without requiring trust between the bodies. Limitations to this decentralized model include reduced control of hospitals or clinics over their patient data because this control is now handed over to the patients. However, this level of control over data can also be customized in a blockchain solution such that hospitals can still retain a level of control that is workable and efficient for their workflows. In addition, patients will have simpler, more efficient, and timely access to their data, thereby enabling better health outcomes because of larger data access for treatments.

## Discussion

### Lack of Flow of Information Back to the Primary Health Care System

The overview of health information flow in British Columbia illustrates that hospital EMRs only send information in one way. There is no direct, timely, and complete flow of information back to the EMR in hospitals. Although the diagnostic imaging and laboratory test result databases work well by allowing different physicians with access to view their patients’ test results, it is often only their past results and not their latest test results. These results could take days to weeks to become accessible in the database. Therefore, patients may still be required to repeat expensive, sometimes even painful, tests when treated by different physicians working in different facilities. In addition, additional information such as consultation notes, diagnoses, or discharge summaries are sent to these provincial databases for record keeping with no intention of sharing it with other hospitals or patients in a retrievable format. Indeed, the sending hospital must keep its hard copy of the notes, as the copy sent to the provincial database is not accessible by frontline health care staff. As a result, patients who are transferred between hospitals receive extremely inefficient care as their file must be physically transferred with them. These manual processes often result in man-made mistakes, misplacement, or missing pages, or even a swap of pages between patients and mistakes with a huge impact when it comes to providing care for sick patients [40]. This issue leads directly to the next issue, the lack of focus on improving frontline care.

### Lack of Focus on Direct Improvement of Patient Care

The entire British Columbia health care data system infrastructure was not built to make frontline patient care better immediately. It was built to collect information and analyze outcomes over time, to provide raw data for research, and to create new standards of care for future patients. Although this is an important goal, it does not help current patients receive better, timely, and evidence-based care. In short, there is no focus and no budget to help current patients.

Given the manner in which the current system is built, hospitals are encouraged to continue using physical notes; digitization of these notes only serves to fulfill the hospital’s legal obligation to send information to government databases. As the complete set of information never comes back in quick-enough turnaround time to inform health care decisions for the same patient, health care professionals would rather keep a complete set of physical notes on hand at the hospital than spend time and resources to change the *status quo*. However, if there is a system that allows health care staff to enter patient notes, diagnoses, and collect test results in real time, with easy accessibility from different institutions when strictly guarding privacy and permissions, there would be a much larger incentive for physicians to change their habits. This would assist health care staff in conforming to a digital health data storage solution, which allows better patient data transfers and can build a more complete patient health profile over time.

This calls for a significant change in how health care data networks should be managed, as both current and future care are equally important and should be taken into consideration in a health care data storage and transmission network.

### Network Latency

Network latency refers to the delay between an order to transfer and the actual transmission of the data. This is a critical issue in health care data networks as information is often needed immediately, and consequences can be dire if there are information mix-ups or delays. Due to the build of the current system, health care staff and personnel cannot transfer real-time, updated digital information to any health care professional who needs it. Doctors write physical notes, and these papers are given to coders who digitize the information month by month. Although any information entered by these coders is seen immediately in the provincial database and the data warehouse, no party will begin processing and analyzing these data until the month cutoff date is reached and the coders declare that the block of monthly information entered is complete and correct. Therefore, as a patient progresses in the hospital, their eHealth records can get very disjointed and information can easily be lost or incorrectly linked over time. This means that there is a significant, unbridgeable gap between finalized digital health information passed onto the government and the most current unprocessed information collected in the hospital. This leads to the conclusion that current EMR systems themselves, and the way they are used by health care professionals, are not optimized for frontline care and should be re-evaluated with the active patient as the focus rather than the focus on data collection.

### Information Silos

Many information silos are shown in Figure 1. Individual hospitals (depicted by dark blue) that do not use the same EMR as other hospitals are only able to send information to each other via fax or physical transfers of papers, and private clinics (depicted by green).

### Barriers to Health Data Digitization in British Columbia

The PITO reports that British Columbia has one of the highest rates of EMR adoption, where 80% of physicians in the province had an operating EMR system [27]. However, this statement does not reflect the optimization of EMR software within a clinic or a hospital. On consultation with the industry experts described above, it became evident that EMR systems in hospitals across the PHSA were using highly outdated methods for information sharing, although EMR systems were deployed and adopted [41]. This implies that systems integration is still severely lacking, and there is inadequate will and incentive to optimize use. Although there is a massive digitization backlog, an adequate integration system that allows interoperability between health care facilities has yet to be identified.

There are notable cost implications with effective integration given that for an EMR system to be useful from the onset, it must be introduced and integrated at every level of care [27]. To provide context, handwritten notes are transcribed to charts that are then transcribed to an EMR. After this point, when a patient is seen at a neighboring hospital within PHSA, sensitive patient information is transferred via hospital courier, irrespective of whether that information has been digitized or not. Therefore, redundancy and inefficiency are blatant, but the financial implications to eliminate paper notes are tremendous [19]. This is a huge barrier to effective digitization efforts.

Furthermore, there is a steep learning curve when adopting a new system entirely [29]. Optimized health data digitization takes time and patience; doctors and health care staff must be in agreement to take on the endeavor to reroute the current *status quo* [19]. However, getting all parties on board and launching such a massive training program takes time. There is a scale-up process that may take years to implement even within a single hospital. Furthermore, given the nature of the private industry in which EMR vendors operate, there are limited regulations and standards that companies comply with across the board [42]. This means that when different facilities take on different vendors as their IT solution proponents, standardization of how the system is used and how it communicates is undetermined until initial adoption [42]. The process by which vendors are selected is a serious factor with political influence that affects interoperability and data digitization.

### Limitations of Blockchain

The personal identity key setup discussed in the Results section begs an important question: what if a user loses their identity key? In traditional blockchain systems, once a user loses their key, their data become completely irretrievable. Essentially, their data are lost forever. This is not acceptable for medical records [15]. Therefore, any future blockchain health care data management system will need to have built-in key retrieval processes that are as secure as the blockchain itself. These protocols have already been invented and are actively in use in other industries, such as payment systems [43]. Possible solutions to handling key retrieval after death can follow current protocols: allowing next of kin or the person with power of attorney the ability to retrieve the key if it is lost or if the original owner is incapacitated.

Another issue that may come to mind is throughput. Health data are seemingly endless, and more data are being generated at a dizzying pace each day, which requires sophisticated software and enormous amounts of computing power to sort through. Low throughput has also been a popular criticism of early blockchains, such as Bitcoin and Ethereum [44-46]. However, poor throughput is a common phenomenon in early and badly constructed blockchains [44]. Many inexperienced coders will put certain processes and data on the chain that should not be on the chain, for example, the actual protocol and private user data. This is one of the major reasons why throughput is assumed to be a common problem (personal communication, blockchain consultant) in addition to old consensus fabrics that simply could not handle high volumes efficiently [44]. In addition, large public chains also have poor throughput, as the number of users often increases exponentially without sufficient server and engineering support. However, a well-constructed private chain has been demonstrated to be able to handle high volumes of transactions. Examples include the University Health Network, which launched a blockchain-based patient consent gateway in 2018, in partnership with eHealth Ontario and IBM. It enables patients to share specific digital medical records with trusted providers and monitor entities’ access to different records [47-50]. VeChain has active solutions for incentive programs, supply chains, logistics solutions, copyright tracking, document management, and smart agriculture [51]. There have also been significant leaps in research in the past few years in the scalability and efficiency of blockchains [44,45,52], which has paved the way for the possibility of a high-throughput health care blockchain.

### Proposed Implementation Plan of Blockchain Solution

#### Viability of Blockchain

The success of a blockchain solution is directly underpinned by the rate of health record digitization. Issues encompassing a lack of flow within the health system, the lack of focus on the primary health system, and network latency are directly related to how quickly patient records are transcribed, coded, digitized, and made available in EMRs. The proposed solution, as outlined below, is built upon the existing cloud-based EMRs that are dependent on health data digitization. Therefore, although blockchain resolves the issue of information silos, it does not bridge the gap between physical and electronic records. The viability of blockchain as a solution for interoperability is contingent on health data digitization.

##### Phase 1: Blockchain in Private Clinics

**Figure 2:**
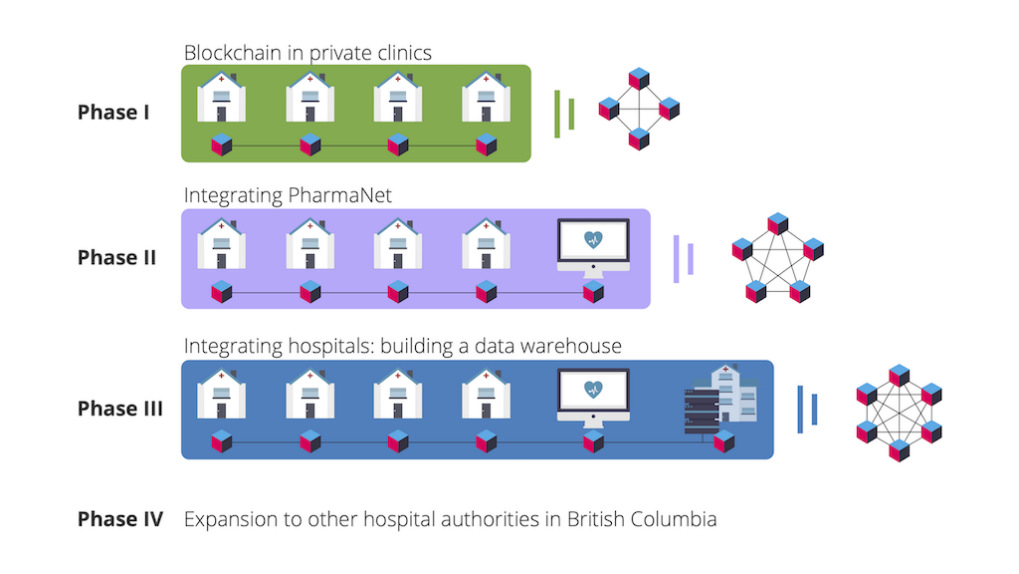
The 4 proposed phases of implementation of blockchain in British Columbia’s health care data management.

First, a suitable blockchain EMR system needs to be built (Figure 2) that will connect all private clinics. The necessary components of a blockchain-based EMR are as follows: the use of ZKPs to execute contracts and transactions on the blockchain, self-sovereign identity framework, a decentralized cloud for data storage, and the permissioned blockchain itself for storage of proofs of interactions. These are described in the following paragraphs.

ZKPs mean that as the blockchain is executing commands or transactions, it does so without knowing about anything that is contained within the files being stored, uploaded, or transferred between nodes [6-8]. In an oversimplified example of a ZKP, an imaginary young person goes to the bar. The imaginary bouncer needs to check their ID to ensure that they are of age before allowing entrance. In this scenario, instead of showing a piece of ID that tells the bouncer exactly how old this person is (and contains a lot of other sensitive information), a ZKP could present a simple, trusted statement to the bouncer that the person is old enough to enter. The main point is that the bouncer never needs to know how old the bar goer is, just that their age satisfies the requirement. This is a special feature of blockchains and it enhances security and privacy because there is minimum revelation and sharing of data between third parties [6-8]. However, attention must be paid to the type of ZKP used in the health care data blockchain. There are some types of ZKPs in use currently that are very inefficient and would not be viable in a health care setting, for example, the Zcash public chain, which uses a zero knowledge protocol called zkSNARK (zero knowledge succinct noninteractive argument of knowledge) [53,54]. However, other types of ZKPs exist and are currently in use in different industries, such as in financial services, risk management, insurance, and supply chain management, demonstrating that there are ZKPs efficient enough to handle large-scale data requests [55,56]. Running times of specific ZKPs are published in academic papers, but the methods of creating efficient ZKPs are not published as they are considered proprietary techniques in nearly all leading blockchain companies. In addition, techniques involving combining hardware with the protocols, such as designing chips that can run certain cryptographic algorithms, are other methods of speeding up running times immensely. These techniques are already in use in many industries, and their viability is widely accepted among blockchain technology experts ([55,56]; personal communication, blockchain consultant).

A private self-sovereign identity framework means that each party in the network has been verified and has a way of confirming their identity each time they access the blockchain, ensuring that other identities are not allowed to access or make any transactions in that network. This allows adherence to Canadian privacy laws regarding health data but also complies with the stricter General Data Protection Regulation from the European Union (EU), which applies whenever an EU citizen’s information is involved. It also allows accurate, private, and efficient retrieval of data from storage.

Regarding cloud data storage, as each node stores its records, it creates decentralized storage of data. Although the nation or province’s health care records will all be retrievable from any node given the correct permissions, there will be no large central database. This is critical as large databases often become an inviting target for hackers; decentralization discourages information breaches.

A permissioned blockchain is a feature that gives the EMR shared trust and immutability functions. A permissioned chain means that parties cannot freely join the network and become a node. They must obtain permission from the other nodes in the network before gaining access to any information shared on the network. Only proofs are stored on the chains, never personal data. However, it can facilitate transfers and keep a record of these transfers on the chain without ever knowing what was in the transferred files, with the help of ZKPs. As it must be a permissioned chain, the type of platform that can be used to build this chain is limited. Ethereum is a permissionless chain and therefore cannot be used; Corda is an option, but its peer-to-peer design will impose significant restrictions on the building of the platform. Hyperledger fabric would be an ideal platform to use, as it is a private chain and its channels are more flexible and allow for more options to be built in (personal communication, blockchain consultant).

In this phase, the nodes in the blockchain will be the doctors or health care staff of the individual clinics, insurers, and regulators.

##### Phase 2: Integrating PharmaNet

Once the individual clinics have been linked in a blockchain EMR network, the next step is to integrate PharmaNet [23]. Individual clinics, especially family doctors, depend on pharmacists to fill prescriptions. Increased communication between pharmacies and clinics would allow monitoring over prescriptions to be filled legally and properly, which could reduce prescription drug fraud.

PharmaNet is an existing network [23]; therefore, further studies are needed to determine how this platform can feed information to the blockchain. Ideally, PharmaNet will transition into a fully blockchain-based network in phases, phasing out the use of their existing network. As this is not always possible, other solutions can include building a data warehouse to store and reformat all information collected from PharmaNet and injecting this into the blockchain via a single node for PharmaNet. However, if PharmaNet wants reciprocal information from clinics, they will need to access this information via the blockchain EMR system. As they use it for their own needs, that is, to retrieve the information they want, they will slowly get accustomed to the blockchain EMR interface, which will help smoothen the transition between PharmaNet and the blockchain PharmaNet. More details on building a data warehouse are presented in the upcoming section on integrating hospitals, as hospitals are likely more resistant to switching EMR systems. This will require a phase-by-phase transition into a blockchain EMR, similar to PharmaNet’s transition, but at a slower pace.

##### Phase 3: Integrating Hospitals—Building a Data Warehouse

In order for a hospital to be added to the blockchain network where the blockchain acts as an external body, a separate data warehouse would need to be constructed to tether the network to the hospital. On the basis of the information flow presented in Figure 1, there are restrictions and privacy limitations on the flow of information from EMR systems to the existing PHSA data warehouse. The technical components of creating an API between existing hospital EMRs or the regional data warehouse that system feeds information into requires permission to be accessed on several levels. According to the industry expert consulted for this work, gaining access or permission to connect to existing infrastructure is challenging because of data governance. Given these limitations, when it becomes relevant for hospitals within the region to join the network for the preliminary purpose of accessing patient information from external clinics as opposed to sharing data externally, a separate database must be developed. This proposed database connects to the hospital EMR and enables an API connection to the blockchain node.

The process of building a data warehouse is a complex and sensitive endeavor itself without considering that it would be a stepping stone to the blockchain. Health Catalyst, a health care data analytics company, proposes a late-binding approach to data warehouse implementation as opposed to the enterprise data model or independent data model. A late-binding approach differs from a solely top-bottom or bottom-up method in that it is more pragmatic and equipped to handle the rapidly changing environments within health care [57]. This method uses features of both enterprise data warehouse and independent data mart, where data are taken from the source system in its most atomic form and transferred into course marts that exist within an enterprise data warehouse. Unlike independent data marts, data are not transformed as soon as they are transferred out of their source; rather, the data are kept in these raw marts in their rawest form [57]. Data are then transferred from the source marts to a data mart where data transformation and binding can occur. This is an incremental model that allows for data binding only when necessary [57]. It eliminates the disadvantages associated with the enterprise data model, which forces one to hammer out the system at the beginning before there is an understanding of what the data can be used for. This model can be thought of as a just-in-time data-binding approach. Decisions are made only about data transformation and binding as needed [57].

##### Phase 4: Expansion to Other Hospital Authorities in British Columbia

Once the blockchain EMR is fully established and functional within all hospitals and the smaller health care facilities in PHSA, this blockchain can be pushed toward the other 6 hospital authorities in British Columbia. PHSA will act as a catalyst and demonstrate the possibility of a controlled, private, yet free flow of health care information for those who require it and have permission to view it. Implementation in other hospital authorities will likely be able to follow the same general steps as implementation in PHSA, but, of course, each authority will have unique challenges and requirements. Therefore, a detailed and full-scale consultation with each stakeholder should be done before making changes to ensure that the transition is smooth and to safeguard the quality and availability of patient care.

#### Conclusions

The exploration of the *status quo* in British Columbia’s health care data management has exposed information silos even within small communities, expensive mistakes, and issues with implementation, and a general unwillingness of parties within a health care system to trust and share data. Other challenges include the potential for security breaches and operational issues in the current EMR infrastructure, although existing EMR systems follow legal security requirements stipulated by the government.

Compared with alternative technologies such as cloud-based solutions, IT solutions, and EMR systems, the blockchain-based solution has the highest potential for solving most of the common challenges in managing health care data. Blockchain offers (1) a plausible system to unite different groups that do not trust each other, (2) decentralized storage to increase security, (3) sovereign identities to give patients and health care facilities secure access to medical records, and (4) most importantly, interoperability to allow the transfer of medical records to anybody who has permission and needs to access them. A blockchain-based solution will change the current infrastructure to a more universal and patient-centric system. The implementation plan will include targeting independent clinics in the PHSA of British Columbia first, then including PharmaNet, which will be followed by local hospitals. After establishing a strong and secure network to demonstrate the benefits that blockchain can bring to health care data management systems, the solution can be expanded to other hospital authorities in British Columbia and in the other provinces in Canada.

Further studies are required to address the limitations of using blockchain in health data management. More work is needed specifically in designing built-in key retrieval processes that are as secure as the blockchain itself as part of the health data management process. The future direction also includes exploring solutions to address the lack of health records digitization, which prevails and underpins health records management as a whole.

## Abbreviations

API: application programming interface
BCMOH: Ministry of Health in British Columbia
BCPHSA: British Columbia’s Provincial Health Services Authorities
CDI: Clinical Diagnostic Imaging
CIHI: Canadian Institute for Health Information
EMR: electronic medical record
IAR: integrated assessment record
IT: information technology
MIT: Massachusetts Institute of Technology
PCSA: point-of-care service application
PHIP: Public Health Improvement Plan
PHSA: Provincial Health Services Authority
PII: personally identifiable information
PITO: Physician Information Technology Office
PLIS: Provincial Laboratory Information Solution
ZKP: zero knowledge proof
